# Screening of Antioxidative Properties and Inhibition of Inflammation-Linked Enzymes by Aqueous and Ethanolic Extracts of Plants Traditionally Used in Wound Healing in Poland

**DOI:** 10.3390/antiox10050698

**Published:** 2021-04-28

**Authors:** Marta Mainka, Monika E. Czerwińska, Ewa Osińska, Maria Ziaja, Agnieszka Bazylko

**Affiliations:** 1 Department of Pharmacognosy and Molecular Basis of Phytotherapy, Warsaw Medical University, Banacha 1, 02-097 Warsaw, Poland; marta.rogowska@wum.edu.pl (M.M.); monika.czerwinska@wum.edu.pl (M.E.C.); 2 Department of Vegetable and Medicinal Plants, Warsaw University of Life Sciences, Nowoursynowska 159, 02-776 Warsaw, Poland; ewa_osinska@sggw.pl; 3 Institute of Physical Culture Studies, Rzeszów University, Cicha 2a, 35-326 Rzeszów, Poland; mziaja@ur.edu.pl

**Keywords:** *Achillea millefolium*, *Arctium lappa*, Asteraceae, hyaluronidase, Lamiaceae, lipoxygenase, reactive oxygen species, *Thymus serpyllum*

## Abstract

A wide range of plant-derived preparations have been used against skin inflammatory disorders and as wound healing agents in traditional medicine. The purpose of the study was to determine the antioxidant activity of aqueous and 70% ethanolic extracts from eleven species of plants traditionally used in Poland to treat inflammatory skin diseases. The ability of extracts to scavenge 2,2-diphenyl-1-picrylhydrazyl radical (DPPH), hydrogen peroxide (H_2_O_2_), and superoxide anion (O_2_^•^^−^), was studied. In non-cellular studies, an analysis of the anti-inflammatory effect on the activity of enzymes, such as hyaluronidase (HYAL) and lipoxygenase (LOX), was also performed. The chemical profiles of the most active extracts were achieved by applying the UHPLC-DAD-MS^n^ method, and the sum of polyphenols in all tested extracts was determined by the colorimetric method with the Folin–Ciocalteu reagent. The scope of the extracts’ influence on enzyme activity was significantly lower than their antioxidant activity. All extracts have shown high activity in free radical scavenging against DPPH. The ethanolic extracts have shown high potential to scavenge H_2_O_2_. The study of composition showed that the main components of the tested extracts were flavonoids, such as luteolin, apigenin, kaempferol, and quercetin derivatives, as well as caffeoylquinic acids, caffeic acid, and its conjugates.

## 1. Introduction

Historically, plant-based natural products were the main source of medicinal preparations available to cure any human diseases [1]. Tea infusions as well as alcoholic extracts from plant materials have long been used to treat skin diseases, insect bites, itching, and scratches. They may also help to treat bruises, tumors, and gouty swellings [2]. In Poland, the water extract is one of the most popular forms of the natural preparation so far, and it is a reason why we tested the activity of selected plant materials in this form. More recently, the practice of evidence-based herbal medicine is getting more attention among patients and physicians [3]. A wide range of plant-derived preparations from species selected for research, including *Achillea millefolium* L. *s. str.* (Asteraceae), *Arctium lappa* L. (Asteraceae), *Arctium minus* (Hill) Bernh. (Asteraceae), *Calendula officinalis* L. (Asteraceae), *Centaurea cyanus* L. (Asteraceae), *Galium aparine* L. (Rubiaceae), *Sambucus nigra* L. (Adoxaceae), *Thymus serpyllum* L. (Lamiaceae), *Taraxacum officinale* F. H. Wigg. (Asteraceae), *Urtica dioica* L. (Urticaceae), or *Viola tricolor* L. *s. str.* (Violaceae), is traditionally recognized as having medicinal value in the treatment of skin disorders [4,5,6]. All these plants are used in folk medicine to treat skin disorders caused by mechanical teasing, itching, acne, burns, and frostbite, and to accelerate the process of wound healing [7]. According to the European Medicine Agency (EMA), from all the plant materials chosen for the research, only *Calendulae flos* and *Millefolii herba* monographs describe the therapeutic indications for their external use in skin disorders. These indications include symptomatic treatment of minor inflammations of the skin, such as sunburn, and mucosal lesions as well as the healing of minor wounds. The data obtained in some studies showed that the oil yarrow extracts had an evident anti-inflammatory property, and application of tested oil extracts on artificially irritated skin in vivo demonstrated the ability to re-establish their optimal pH and hydration of skin to the values measured prior to the irritation [8]. In the case of *Arctii lappae radix* and *Violae herba,* their traditional use in the treatment of seborrhoeic skin conditions is mentioned in the EMA monographs. Other plant materials, like *Centaurea cyanus* L. (flowers), *Galium aparine* L. (herb), *Urtica dioica* L. (leaves), *Taraxacum officinale* F. H. Wigg. (leaves), *Thymus serpyllum* L. (herb), as well as *Sambucus nigra* L. (leaves), seem to be less known in such an indication in spite of some data on their role in traditional treatment of skin disorders.

Skin is the body organ that provides an interface between the environment and organism. It is permanently exposed to environmental, chemical, and physical pollutants, including ionizing and UV radiation, as well as car exhaust and industrial resources. These environmental toxicants are oxidative agents or lead to the production of reactive oxygen species (ROS), which are usually short-lived entities generated even in normal aerobic metabolism [9]. In particular, ROS include singlet oxygen (^1^O_2_), superoxide anion (O_2_^•^^−^), H_2_O_2_, and the hydroxyl radical (OH^•^). The stepwise sequential univalent reduction of O_2_ led to the formation of O_2_^•^^−^, H_2_O_2_, and OH^•^. Free radical reactions are usually chain reactions, like the Fenton reaction, which generate the subsequent radicals as reaction products [10]. The main role of ROS is to kill and destroy invading microorganisms and/or degrade damaged tissue structures. However, the imprecise targeting of ROS can induce oxidative stress in adjacent normal cells, leading to enhancement of pathologic processes. Uncontrolled release of ROS is involved in pathogenesis of a wide range of human skin disorders such as cutaneous neoplasia and other age-dependent disorders [11,12]. In addition, the photoaging effects of sunlight are closely related to the generation of ROS, which influence the activation of NF-κB and mitogen-activated protein kinases (MAPK) such as ERK, JNK, and p38 kinases responsible for activation of transcription factor AP-1 [9]. These factors regulate the genes that are involved in the pathogenesis of inflammation. Therefore, the plant-derived preparations seem to be an important source of free radical scavengers that protect against inflammation and eliminate environmental ROS or their byproducts from the skin surface. Additionally, they support the human defense system against oxidative stress, which is composed of glutathione, glutathione peroxidases, glutathione reductase, glutathione *S*-transferases, superoxide dismutases, catalases, and quinone reductase.

Therefore, the main aim of the study was to screen and compare the activity of extracts traditionally used to treat skin disorders as well as to select the plant extract regulating the wound healing process through the modulation of the inflammatory response. The effect of aqueous and ethanolic extracts on the activity of enzymes (hyaluronidase-HYAL and lipoxygenase-LOX) responsible for prolongation of the inflammatory state was performed in cell-free systems. Taking into consideration the role of ROS in the development of inflammation, in the present study we have investigated the scavenging activity against superoxide anion (O_2_^•^^−^), hydrogen peroxide (H_2_O_2_), and synthetic radical 2-diphenyl-1-picrylhydrazyl (DPPH) in cell-free systems by the polyphenols-standardized extracts [13,14]. The chemical compositions of the most active extracts were studied using the UHPLC-DAD-MS^n^ method.

## 2. Materials and Methods

### 2.1. Chemicals and Reagents

Allopurinol, ascorbic acid, bovine serum albumin (BSA), 2,2-diphenyl-1-picrylhydrazyl radical (DPPH), horseradish peroxidase (HRP), hydrogen, peroxide (H_2_O_2_), linoleic acid (LA), luminol, hydrochloride, hyaluronic acid (HA), hyaluronidase from bovine testes (BTH), nitrobluetetrazolium (NBT), nordihydroguaiaretic acid (NOR), phosphate buffered saline (PBS) PAA Laboratories (Pasching, Austria), xanthine (X), and xanthine oxidase (OX) were purchased from Sigma-Aldrich Chemie GmbH (Steinheim, Germany). Acetic acid, boric acid, chloroform, ethanol 96° p.a., Folin–Ciocalteu reagent, hydrochloric acid, methanol p.a., sodium acetate, sodium carbonate, sodium chloride, sodium dihydrogen phosphate, and sodium hydroxide were purchased from POCH (Gliwice, Poland). Acetonitrile and formic acid for UHPLC were purchased from Sigma-Aldrich (St. Louis, MI, USA). Solvents used for the UHPLC analysis were UHPLC grade. Deionized water was obtained using a Milli-Q Plus MILLIPORE, Billerica, Massachusetts USA (18.2 MΩ cm). Castalagin (purity > 95%) was isolated from *Lythrum salicaria* L. herb in the Department of Pharmacognosy and Molecular Basis of Phytotherapy, Medical University of Warsaw, Poland [15].

### 2.2. Plant Material and Extract Preparation

Plant material was harvested from a cultivated field belonging to the Warsaw University of Life Sciences in the central region of Poland (Masovia) and the south-east region of Poland (the Subcarpathian province) during the flowering period from April to October in 2016 (52°9′42.2” N 21°6′22.211” E; 49°54′36.35” N 21°32′47.514” E), and next dried at room temperature in the shade. Botanical identification was performed by Prof. Ewa Osińska from the Department of Vegetable and Medicinal Plants, Warsaw University of Life Sciences, Poland, and Dr. Maria Ziaja from the Institute of Physical Culture Studies, Rzeszów University, Poland. A specimen of the raw material (Am15092016, All23072016, Alr24072016, Co06082016, Cc18052016, Ga24062016, Vt22082016, Ths13062016, To11072016, Ud14092016, Sn30062016, Amr08082016, Aml05082016) is available in the drug collection of the Department of Pharmacognosy and Molecular Basis of Phytotherapy, Medical University of Warsaw, Poland.

The raw material was grounded using an IKA MZO electric grinder (IKA-WERKE, Staufenim Breisgau, Germany) and extracted. Two hundred milliliters of water was added to an accurately weighed 10 g portion of raw material, and the sample was kept under reflux in a water bath (temperature appx. 90 °C) for 1 h. The process was repeated three times using the same volume of solvent, and then the extract was filtered under a vacuum through filter paper with an average speed of filtration on a Büchner funnel. The 70% (*v*/*v*) ethanolic extract was prepared in the same conditions. The ethanolic extract was evaporated to dryness in the LABORANTA 4000 WB Germany Heidolph evaporator, and the residue was suspended in 200 mL of water. To remove a significant amount of chlorophyll from the ethanolic extract, a liquid–liquid extraction with chloroform (three times with 200 mL) was performed. The water residue extracts were lyophilized using a laboratory freeze-dryer, Cryodos (Telstar, Terrassa, Spain). After lyophilisation, the residues of extracts were separately pulverised, carefully mixed, and stored at 4 °C. The timetable for preparing the extracts and conducting the experiments can be found in the supplementary materials (Table S1).

### 2.3. Biological Activities

#### 2.3.1. Evaluation of Enzyme Activity Inhibition in Cell-Free Systems

Anti-hyaluronidase activity was measured using the turbidimetric method (USP XXII-NF XVII (1990) 644–645, United States Pharmacopoeia Convention, Inc., Rockville, MD) [16] modified to a 96-well microtiter plate volume (500 µL) by Piwowarski et al. (2011) [17]. The extracts were tested at concentrations of 50, 150, 300, and 500 µg/mL. As a positive control, castalagin was used at a concentration of 10 µg/mL (10.7 µmol/L). Inhibition of lipoxygenase (LOX) was determined by the method according to the SIGMA Enzymatic Assay of Lipoxidase (EC 1.13.11.12), which was modified to a 96-well microliter plate volume (200 µL) based on the previous research [18]. The extracts were tested at concentrations of 100, 200, 300, and 500 µg/mL. As a positive control, NOR was used at a concentration of 250 µg/mL (826.8 µmol/L). The percentages of enzyme inhibition were calculated in comparison to the control without the test extracts. To evaluate whether extracts affected the O_2_^•^^−^ generation by direct interaction with xanthine oxidase, the enzyme activity was determined using a xanthine–xanthine oxidase system by monitoring the uric acid formation at 295 nm [19]. As a positive control, allopurinol was used at a concentration of 25 µg/mL (183.7 µmol/L). Three independent experiments were carried out in triplicate samples in all the used methods.

#### 2.3.2. Evaluation of Free Radical Scavenging in Cell-Free Systems

All determinations were made using 96-well plates and were measured in a microplate reader, SYNERGY 4 (BioTek, Winooski, VT, USA). Each lyophilized extract was dissolved in 50% ethanol (DPPH scavenging assay) or in PBS. The ability to scavenge DPPH radical was examined using the method of Choi et al. (2002). The extracts were tested at concentrations of 10, 20, 50, 150, and 250 µg/mL. Ascorbic acid was used as a positive control at a concentration of 7 µg/mL (39.7 µmol/L). The scavenging of O_2_^•−^ was tested using a xanthine–xanthine oxidase system with the NBT reduction method as described by Choi et al. (2002) [20] and modified by Kiss et al. (2010) [21]. The extracts were tested at concentrations of 5, 10, 25, 75, and 125 µg/mL. Ascorbic acid was used as a positive control at a concentration of 12.25 µg/mL (69.5 µmol/L). The scavenging of H_2_O_2_ was examined using the method of O’Dowd et al. (2004) [22] modified by Kiss et al. (2010) [21]. Hydrogen peroxide scavenging was performed with horseradish peroxidase in the presence of a H_2_O_2_ solution, and the chemiluminescence of luminol was measured. The extracts were tested at concentrations of 2.5, 5, 15, 25, and 50 µg/mL. Ascorbic acid was used as a positive control at a concentration of 3 µg/mL (17.0 µmol/L). The percentages of scavenging activity were calculated in comparison to the control without the test extracts. Three independent experiments were carried out in triplicate in all the used methods.

### 2.4. Phytochemical Analysis

#### 2.4.1. Total Phenolic Content

The tested extracts were chemically characterized by determining the sum of polyphenols with the colorimetric method using the Folin–Ciocalteu reagent. This assay was performed in a 96-well plate. The sample of tested extract (stock solution 1 mg/mL, 40µL), a 10% (*v*/*v*) Folin-Ciocalteu reagent (105 µL), and 1 molar solution of Na_2_CO_3_ (85 µL) were mixed at a speed of 420 RPM during a 15 min incubation at 45 °C (Microplace Shaker DTS-2, Elmi), and next the absorbance of each well at 765 nm was read in a microplate reader, SYNERGY 4 (BioTek, Winooski, VT, USA) [23].

#### 2.4.2. UHPLC-DAD-MS^n^ Analysis

The HPLC Ultimate 3000 system (Dionex-Thermoscientific, Sunnyvale, CA, USA) equipped with a dual low-pressure gradient pump, an autosampler, a column compartment, a diode array detector, and an AmaZon SL ion trap mass spectrometer with an ESI interface (Bruker Daltonik GmbH, Bremen, Germany) was used to perform the analysis. HPLC analyses were carried out on a reversed-phase Zorbax SB-C_18_ column (150 × 2.1 mm, 1.9 µm; Agilent, Santa Clara, CA, USA). The mobile phase: (A) 0.1% HCOOH in H_2_O; (B) 0.1% HCOOH in MeCN; 0–60 min, 10–60% B; flow 0.2 mL/min; and column temperature 25 °C. The column was equilibrated for 10 min between injections. UV–VIS spectra were recorded over a range of 200–600 nm, chromatograms were acquired at 254, 325, and 350 nm. The LC eluate was introduced directly into the ESI interface without splitting. The nebulizer pressure was 40 psi, dry gas flow was 9 L/min, dry temperature was 300 °C, and capillary voltage was 4.5 kV. The analysis was carried out using a scan from *m*/*z* 200–2200. The compounds were analyzed in a negative ion mode. The MS^n^ fragmentation was obtained for the two most abundant ions at the time. The detection of neutral losses was set for the sugars or phenolic acids (162, 132, 176). The identification of compounds was performed based on a comparison with literature data [24,25,26,27] and comparison with chemical standards.

### 2.5. Statistical Analysis

The results were expressed as a mean ± standard deviation (SD) of the indicated number of experiments. The SC_50_/IC_50_ values of the tested extracts, expressed in µg/mL, were calculated based on concentration–activity curves. To calculate the SC_50_/IC_50_ values, in cases where the relationship between activity and concentration was not linear, logarithmic functions were used; only in the case of lipoxygenase the linear function was used for calculations (LOX—the graphs for the most active extracts were provided as supplementary materials, Figure S1). The statistical significance of the differences between the means was established by ANOVA with Tukey’s (comparison between the tested extracts activity) or Dunnett’s (comparison between the tested extracts and a control activity) post hoc tests. All analyses were performed using Statistica 13. The differences between groups were considered to be significant when the *p* value was < 0.01.

## 3. Results and Discussion

Inflammation is part of the non-specific immune response that occurs in reaction to any type of bodily injury. Under normal conditions, the inflammatory process is self-limiting. In some disorders, it becomes continuous and might lead to chronic inflammatory diseases [28]. In skin disorders, prolonged inflammation is detrimental and may result in deregulated differentiation as well as an activation of keratinocytes, accelerating the process through the normal stages of wound healing [29]. Effective repair requires communication and interplay between many different cell types. This process is precisely controlled and regulated at multiple levels [30]. For instance, lipoxygenases are the major enzymes involved in the inflammatory process. They metabolize the arachidonic acid produced to leukotrienes [31]. Other enzymes significant for wound healing are hyaluronidases, which degrade hyaluronan—a major constituent of the extracellular matrix of the skin, joints, eyes, and many other tissues and organs. Furthermore, hyaluronidases play a key role in each phase of wound healing by stimulating cell migration, differentiation, and proliferation as well as regulating extracellular matrix organization and metabolism [32]. Antioxidant activity against superoxide anion (as a source of free radical—the xanthine–xanthine oxidase system was used) may partly be due to enzyme activity inhibition, and this effect was also tested. Reactive oxygen and nitrogen species also cause degradation of high-molecular-weight hyaluronan, an anti-inflammatory extracellular matrix component [33]. The reactive oxygen/nitrogen-species scavenging potential and enzyme inhibitory activity of plant materials play a crucial role in the protection against skin aging as well as skin disorders. For all the tested extracts, anti-hyaluronidase activity (Table 1) was examined in the concentration range 50 to 500 µg/mL. The most active extract, *Serpylli herba* aqueous extract (Th H_2_O), at a concentration of 150 µg/mL showed the 71.7 ± 4.9% inhibition of hyaluronidase activity. The inhibition was dose-dependent, and the IC_50_ (substance/extract concentration eliciting 50% of the maximum inhibition) value was 118.1 ± 7.1 µg/mL. The activity of the tested extracts was much lower than in the case of castalagin at a concentration of 10 µg/mL (10.7 µmol/L, 98 ± 0.34%). To the best of our knowledge, an inhibition of hyaluronidase activity only in the case of preparations from *Arctium lappa* roots and *Calendula officinalis* flowers in cell-free systems was determined [34,35]. Our research confirmed the above results regarding very low hyaluronidase inhibitory activity of extracts from these plant materials.

Four out of the 26 extracts tested against the 5-LOX enzyme activity (Table 1) showed inhibition activity higher than 50% (IC_50_ range 297 to 461 µg/mL). These were *Urticae herba* aqueous extract (U H_2_O) and *Millefolii herba* aqueous extract (K H_2_O) at a concentration of 500 µg/mL, whereas the aqueous and ethanolic Th extracts were even active at lower concentrations of 300 µg/mL and 400 µg/mL. The ethanolic and aqueous extracts from Th had the highest activity: at a concentration of 500 µg/mL there was approximately 90% inhibition of LOX activity. Almost all the tested extracts showed statistically significant lower activity than the control nordihydroguaiaretic acid at a concentration of 250 µg/mL (826.8 µmol/L, 91 ± 1.67%). There were no significant differences between Th extracts (ethanolic and aqueous) at a concentration of 500 µg/mL and the control. To the best of our knowledge, among the chosen species only extracts from *Arctium lappa* (roots, leaves), *Achillea millefolium* (leaves), *Calendula officinalis* (leaves, flowers), *Urtica dioica* (leaves), and *Taraxacum officinale* (leaves) were tested for a lipoxygenase inhibitory activity in cell-fee systems [31,34,36,37]. Chagas-Paula et al. (2015) [31] showed that IC_50_ for *A. millefolium, C. officinalis*, and *T. officinale* was higher than 50 µg/mL. Our results did not differ from the results of other researchers, indicating weak ability of the above-mentioned plant materials to inhibit lipoxygenase. The found weaker activity of the tested extracts compared to the results of other research groups may result from different collection sites, different preparation of extracts, and other methods used for determination.

In this research, the highest DPPH radical scavenging activity was reported for Th EtOH extract with an SC_50_ (substance/extract concentration eliciting 50% of the maximum radical scavenging) value 15.0 ± 2.09 µg/mL (Table 2). There were no significant differences between the most active extracts: *Arctii lappae folium* aqueous extract (All H_2_O), *Arctii lappae folium* ethanolic extract (All EtOH), K EtOH, and Th EtOH, in the concentration range 150 to 250 µg/mL. However, all of them showed significantly higher activity than the control ascorbic acid at a concentration of 7 µg/mL (39.7 µmol/L, 70 ± 4.42%). Therefore, the Th extracts were strong radical scavengers and good natural antioxidants, which is consistent with the previous reports. Studies conducted by Kindl et al. (2015) [38] have shown an even higher SC_50_ value 6.01 ± 0.44 µg/mL for 70% ethanolic extract from *Serpylli herba*. Results of scavenging activity on DPPH of all tested extracts are presented in Table 2.

Superoxide anion is an extremely reactive radical and it is a biological product in reducing molecular oxygen [39]. In this assay, the highest O_2_^•−^ radical scavengers were Th H_2_O and Th EtOH extracts, with SC_50_ values 20.4 ± 6.10 µg/mL and 13.6 ± 3.45 µg/mL, respectively (Table 2). There were significant differences between the tested extracts at a concentration of 75 µg/mL and the control ascorbic acid at a concentration 12.25 µg/mL (69.5 µmol/L, 50.28 ± 3.14%). Ethanolic and aqueous extracts of All, K, M, S (*Sambuci nigrae folium*), and Th have shown significantly higher activity than ascorbic acid. Some results reported that phenolic compounds, such as flavonoids, are known to possess high O_2_^•^^−^ scavenging abilities [40]. Thus, these results indicate that the Th extract, which contains approximately 21% of total phenols content (Table 3), effectively scavenges ROS and can protect against oxidative damage. In another study, the results of in vitro antioxidant data showed lower O_2_^•−^ scavenging activity (SC_50_ = 2060 µg/mL) than our results (SC_50_ = 218 µg/mL) for the aqueous extract from *A. lappa* root [41]. Moreover, in our study, the extracts prepared from leaves showed even more relevant activity (All H_2_O SC_50_ = 26.52 ± 5.22 µg/mL, All EtOH SC_50_ = 35.19 ± 6.30 µg/mL) than Alk (*Arctii lappae radix)* extracts. Activity of the extracts in a xanthine–xanthine oxidase system was related only to the scavenging effect against O_2_^•−^, and they did not inhibit the enzyme activity. Results of scavenging activity on superoxide anion of all tested extracts are presented in Table 2.

For the H_2_O_2_ assay, three of the most active plant materials were Th, M, and All. The ethanolic extracts showed stronger scavenging activity (SC_50_: 9.41 ± 0.71, 13.10 ± 1.59, 12.28 ± 1.01 µg/mL for Th, M, All, respectively) than aqueous extracts (SC_50_: 10.87 ± 1.04, 14.69 ± 1.25, 17.92 ± 1.84 µg/mL for Th, M, All, respectively). The Th EtOH extract (SC_50_: 9.41 ± 0.71 µg/mL) showed the highest scavenging activity out of all tested extracts. There were no significant differences between the most active extracts at a concentration of 250 µg/mL (All, Th, M ethanolic and aqueous) and control ascorbic acid at a concentration of 3 µg/mL (17.0 µmol/L, 99 ± 0.43%). The results obtained for U EtOH extract are comparable with previously performed research in which the *U. dioica* ethanolic extract at a concentration of 100 µg/mL showed the 83.3% of scavenging activity [42]. Results of scavenging activity on H_2_O_2_ of all tested extracts are presented in Table 2.

The tested extracts were chemically characterized by determining the sum of polyphenols with the colorimetric method using the Folin–Ciocalteu reagent. The lowest content of polyphenols was 2.56 ± 0.15 and 2.57 ± 0.24 (% ± SD) (Table 3) in the aqueous and ethanolic Alk extracts, respectively. The difference between extracts was not statistically significant. The highest content of polyphenols was 20.61 ± 1.13 and 21.25 ± 2.9 (% ± SD) (Table 3) in the aqueous and ethanolic Th extracts, respectively. In addition, the difference between extracts was not statistically significant. Kindl et al. (2015) [30] have recently established that the content of flavonoids in the 70% ethanolic Th extract was 0.40 ± 0.006 (% ± SD), whereas Stojanovic et al. (2012) [43] showed that total phenolic content in the aqueous Th extract was 2.01 ± 0.02 mg/g of gallic acid equivalents in dry weight of extract.

**Figure 1:**
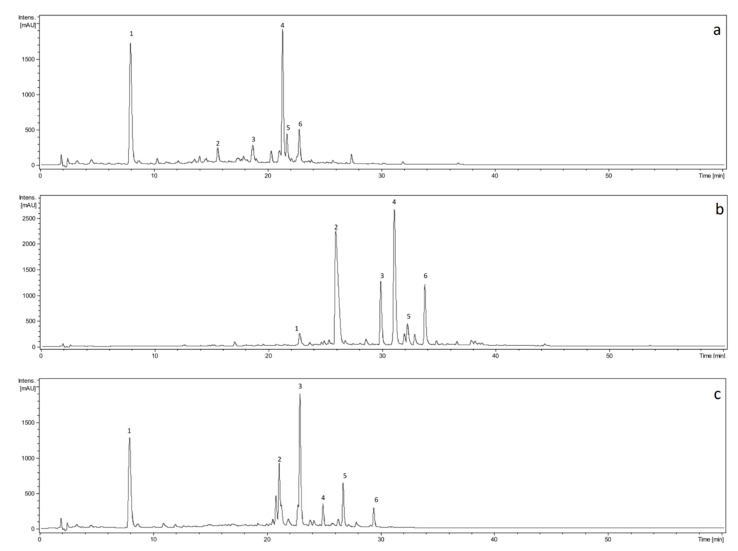
UHPLC-DAD chromatograms of ethanolic-aqueous extracts of (**a**) *Millefolii herba* ethanolic extract (K EtOH), (**b**) *Serpylli herba* ethanolic extract (Th EtOH), and (**c**) *Arctii lappae folium* ethanolic extract (All EtOH), detected at 325 nm.

A study of the composition of the most active extracts showed that the main components (compounds with UV absorption) were flavonoids and caffeic acid conjugates. Flavonoids, in particular luteolin, apigenin, and quercetin derivatives, were detected in K EtOH and Th EtOH extracts (Figure 1, Table 4). More interesting is the fact that among caffeic acid conjugates in the extracts of yarrow herb (K, Figure 1, Table 4) and burdock greater leaf (All, Figure 1, Table 4), mainly caffeoylquinic acids derivatives were observed, while in the extract of the wild thyme herb (Th, Figure 1, Table 4), dimer and trimers of caffeic acid were detected. Our results were consistent with previous studies [24,25,26,27]. The presence of a few caffeoyl moieties in the structures of salvianolic acids found in Th extracts determined the reactivity in the Folin–Ciocalteu reaction in comparison with the phenolic acids found in All extracts. However, it is worth noting that the extraction procedures significantly determine the extracts’ composition [44]. On the other hand, the presence of other compounds, which influence scavenging activity, under the limit of detection or even not detectable with HPLC-DAD-MS^n^, cannot be excluded. The discrepancies between total polyphenol content and the scavenging activities of other tested extracts, such as Calendulae flos and Arctii lappae folium, might result from the presence of specific components for these plants, such as oleanane type saponins [45] or lignans [46], respectively. Therefore, they are likely to enhance the scavenging activity of polyphenol. In addition, polyphenols identified in the most active extracts were chemical substances with proven antioxidant and anti-inflammatory properties. Therefore, these constituents might affect the activity of extracts in a significant manner. Antioxidant and anti-inflammatory properties are demonstrated by derivatives of phenolic acids, such as caffeoylquinic acids, and their succinic and malonyl esters, identified in All EtOH and K EtOH [47], as well as rosmarinic acid and salvianolic acids, which were found in Th EtOH [48,49]. In addition, compounds from the group of flavonoids, derivatives of apigenin, quercetin, and luteolin, present in K and Th extracts, apart from other activities, exert the free radical scavenging effect and anti-inflammatory activity confirmed by previous research [50]. Moreover, the mentioned compounds, in particular derivatives of phenolic acids and flavonoids, also have a documented activity in external use in skin diseases [51,52], and therefore the plant-origin preparations rich in these compounds were of interest to the presented research.

In the present study, the screening of free radical scavenging activity in non-cellular systems was performed. We believe that plant-derived preparations actively enhance the defense system against oxidative stress in skin, in particular via potential reactivity with environmental oxidative toxins. In this case, their external availability allowed us to suspect their utility without epidermal permeability. However, the role of tested extracts and their constituents on the cellular oxidative systems, including NADPH oxidases, still requires elucidation. In cells, the mitochondrial respiratory chain is both the major source of intracellular ROS generation and a key target for the damaging effects of ROS. As a consequence of excessive lipid peroxidation and DNA damage, cell death occurs [53]. On the other hand, in the case of infection, an oxidative burst of neutrophils constitutes the first line of defense against pathogens. NADPH oxidase is a crucial enzyme that communicates during the host responses to a wide variety of viral- or bacterial-derived stimuli [54]. A wide range of polyphenols themselves or polyphenol-rich extracts were proved to inhibit NADPH oxidase [55] or monoamine oxidase [56,57]. Therefore, based on the screening of free radical scavenging properties in addition to the significant number of polyphenols, the active extracts are worth further investigation, in particular in ex vivo models of oxidative enzymes.

## 4. Conclusions

In conclusion, the scope of the extracts’ influence on enzyme activity was significantly lower than their antioxidant activity—especially in the case of the 70% ethanolic extracts’ effect on the hyaluronidase activity. With regards the aqueous extracts, the Th extract from the aerial parts showed the most significant inhibition of hyaluronidase activity. Therefore, the aqueous extracts and the 70% ethanolic extracts from *Serpylli herba* were the most active and they had the highest total content of polyphenols, which can partly be an explanation of their activity. On the other hand, the activity of the extracts may also be influenced by the qualitative composition of the polyphenols. In addition, other compounds in the extracts may play a role, especially in the case of alcoholic extracts, which may result in differences in the activity of water and alcoholic extracts. The activity of extracts from the *Millefoli herba* and *Arctii lappae folium* should not be neglected. The K and All extracts showed high activity and were usually among the three most active extracts. Plant materials are a rich source of compounds with a meaningful potential for therapeutic use in wound care. For this reason, it is important to determine the activity of the selected plant material at different stages of the wound healing process. This research attempted to indicate the beneficial roles of and garner scientific support for medicinal or traditional plant-derived preparations, which are very common in countries of central Europe particularly. It is worth noting that the research established the activity of some plant materials, such as herb of *Galium aparine* and leaves of *Sambucus nigra*, which have not been widely studied to date. The obtained results are encouraging for further studies of the most active extracts.

## Figures and Tables

**Table 1:** The inhibitory effects on lipoxygenase (LOX) and hyaluronidase (HYAL) activity of the tested extracts. IC_50_ (subScheme 50 of the maximum inhibition) for all the tested extracts was higher than 100 µg/mL. ^##^—the most active extract/extracts (*p* < 0.001), ^#^—extracts with higher activity than the others but weaker than the most active ones (*p* < 0.01).

Extract		Anti-LOX (% ± SD)					Anti-HYAL (% ± SD)			
		(µg/mL)								
		100	200	300	400	500	50	150	300	500
*Arctii lappae folium* (All)	H_2_O	8.10 ± 0.35	20.85 ± 0.78 ^#^	27.66 ± 1.06 ^#^	35.93 ± 0.93	46.06 ± 1.14	2.19 ± 1.38	1.94 ± 0.83	2.59 ± 1.00	5.52 ± 0.95
	EtOH	6.89 ± 0.75	14.61 ± 0.40	19.84 ± 0.90	33.69 ± 2.08	39.89 ± 1.07	3.46 ± 3.56	1.23 ± 1.13	4.76 ± 0.71	4.16 ± 1.56
*Arctii lappae radix* (Alk)	H_2_O	1.26 ± 0.20	1.87 ± 0.32	5.37 ± 0.33	7.02 ± 0.45	8.63 ± 0.54	1.42 ± 1.14	6.33 ± 0.94	7.07 ± 1.49	8.68 ± 2.70
	EtOH	0.50 ± 0.23	2.03 ± 0.44	3.01 ± 0.55	5.02 ± 1.09	7.08 ± 1.33	1.75 ± 1.05	7.92 ± 5.18 ^#^	2.53 ± 0.08	3.30 ± 0.34
*Arctii mini folium* (Aml)	H_2_O	13.71 ± 2.10 ^##^	17.86 ± 2.72	19.05 ± 2.88	21.52 ± 1.41	22.65 ± 0.99	0.55 ± 0.15	0.64 ± 0.32	1.31 ± 1.03	2.52 ± 0.55
	EtOH	10.69 ± 1.91	13.64 ± 1.26	16.02 ± 0.55	21.81 ± 1.43	27.85 ± 2.47	0.45 ± 0.21	1.76 ± 0.76	1.72 ± 0.52	2.31 ± 0.86
*Arctii mini radix* (Amk)	H_2_O	7.36 ± 0.58	9.08 ± 0.38	12.95 ± 1.61	20.38 ± 0.73	26.61 ± 0.87	2.99 ± 1.68	4.29 ± 1.75	17.35 ± 0.83 ^#^	46.26 ± 0.81 ^#^
	EtOH	3.96 ± 0.57	9.12 ± 0.62	13.87 ± 0.77	15.69 ± 2.01	21.18 ± 0.88	0.59 ± 0.29	3.99 ± 1.45	4.85 ± 2.22	8.71 ± 2.35
*Calendulae flos* (C)	H_2_O	5.80 ± 0.86	5.69 ± 0.32	8.04 ± 0.64	9.12 ± 0.80	10.96 ± 0.81	0.48 ± 0.66	1.31 ± 1.75	1.80 ± 2.11	2.24 ± 0.79
	EtOH	2.70 ± 0.58	2.35 ± 0.41	4.32 ± 1.69	5.81 ± 0.62	6.63 ± 0.65	0.95 ± 0.83	1.52 ± 0.27	3.64 ± 0.52	5.85 ± 0.32
*Centaureae flos* (B)	H_2_O	5.71 ± 0.65	13.87 ± 1.13	20.09 ± 1.30	22.80 ± 2.08	27.44 ± 2.02	2.11 ± 0.81	2.24 ± 1.65	3.34 ± 1.20	3.09 ± 1.46
	EtOH	3.64 ± 0.24	7.89 ± 0.53	16.16 ± 0.70	23.26 ± 0.90	26.04 ± 1.02	1.43 ± 0.71	3.34 ± 1.53	7.58 ± 1.72	12.05 ± 2.78
*Galii aparinae herba* (P)	H_2_O	7.87 ± 0.44	13.63 ± 0.58	20.22 ± 1.07	26.48 ± 1.45	30.61 ± 0.97	0.87 ± 0.72	2.94 ± 0.76	2.94 ± 0.75	5.25 ± 1.8
	EtOH	3.26 ± 0.19	7.53 ± 0.37	12.15 ± 0.96	19.26 ± 2.46	27.88 ± 2.14	2.98 ± 1.41	2.45 ± 1.06	2.44 ± 1.05	4.73 ± 2.45
*Millefolii herba* (K)	H_2_O	14.94 ± 0.40 ^##^	20.23 ± 1.03 ^#^	33.77 ± 0.85 ^#^	47.27 ± 1.81 ^#^	52.05 ± 4.39 ^#^	0.45 ± 0.32	1.65 ± 0.39	1.90 ± 0.65	3.01 ± 0.57
	EtOH	6.03 ± 0.19	22.07 ± 0.38 ^#^	33.16 ± 1.50 ^#^	42.05 ± 3.98 ^#^	46.49 ± 1.08	0.46 ± 0.23	1.02 ± 0.24	2.78 ± 0.84	2.81 ± 0.52
*Sambuci nigrae folium* (S)	H_2_O	4.92 ± 0.30	10.33 ± 0.55	12.15 ± 1.14	12.66 ± 0.85	21.69 ± 1.42	0.86 ± 0.81	2.30 ± 1.29	2.39 ± 2.62	3.52 ± 2.94
	EtOH	4.69 ± 0.48	6.30 ± 0.24	8.40 ± 0.70	13.45 ± 1.35	19.57 ± 1.96	0.24 ± 0.29	0.88 ± 1.26	3.46 ± 2.13	2.92 ± 1.49
*Serpylli herba* (Th)	H_2_O	11.94 ± 1.22 ^#^	26.18 ± 0.71 ^##^	53.46 ± 0.75 ^##^	70.83 ± 5.15 ^##^	90.58 ± 4.96 ^##^	8.41 ± 0.48	71.71 ± 4.91 ^##^	90.44 ± 3.70 ^##^	98.77 ± 1.50 ^##^
	EtOH	8.29 ± 0.53	24.37 ± 2.11 ^##^	43.73 ± 3.01 ^#^	65.58 ± 3.65 ^##^	90.49 ± 2.27 ^##^	8.24 ± 2.31	8.24 ± 2.31	16.63 ± 4.02 ^#^	28.69 ± 2.48
*Taraxaci herba* (M)	H_2_O	12.21 ± 1.01 ^#^	18.99 ± 1.15	25.06 ± 1.09	19.97 ± 1.28	28.14 ± 1.00	0.36 ± 0.25	2.99 ± 1.50	11.83 ± 3.57	42.28 ± 3.18 ^#^
	EtOH	4.22 ± 0.50	7.13 ± 0.57	9.49 ± 0.25	17.60 ± 1.27	27.42 ± 1.36	1.43 ± 0.80	1.96 ± 0.88	2.87 ± 0.89	3.29 ± 0.83
*Urticae herba* (U)	H_2_O	12.75 ± 0.55 ^#^	26.49 ± 1.4 ^##^	38.55 ± 1.95 ^#^	49.61 ± 1.56 ^#^	60.18 ± 5.58 ^#^	0.82 ± 0.55	2.02 ± 0.91	8.22 ± 3.62	9.77 ± 1.45
	EtOH	4.90 ± 0.46	11.92 ± 0.61	18.70 ± 1.94	25.09 ± 1.22	33.16 ± 4.26	1.68 ± 0.64	2.06 ± 0.52	2.18 ± 0.93	2.18 ± 0.93
*Violae herba* (Vt)	H_2_O	2.53 ± 0.10	6.25 ± 0.54	8.91 ± 0.47	17.16 ± 1.37	24.53 ± 1.54	2.57 ± 0.48	5.21 ± 0.86	12.37 ± 2.76	15.71 ± 4.56
	EtOH	3.33 ± 0.77	5.08 ± 0.67	8.64 ± 0.65	17.71 ± 1.79	19.59 ± 2.22	1.56 ± 1.37	2.23 ± 2.85	4.62 ± 1.88	4.73 ± 2.09

**Table 2:** Scavenging effects of the tested extracts on 2,2-diphenyl-1-picrylhydrazyl (DPPH), hydrogen peroxide (H_2_O_2_), and superoxide anion radicals (O_2_^•−^) generated by the OX/X system. NA denotes a SC_50_ (substance/extract concentration eliciting 50% of the maximum radical scavenging) value higher than 150 µg/mL. ^##^—the most active extract/extracts (*p* < 0.001), ^#^—extracts with higher activity than the others but weaker than the most active ones (*p* < 0.01).

Extract		DPPH (% ± SD); SC_50_ (µg/mL ± SD)					H_2_O_2_ (% ± SD); SC_50_ (µg/mL ± SD)					O_2_·^−^ (% ± SD); SC_50_ (µg/mL ± SD)				
		(µg/mL)														
		10	20	50	150	250	2.5	5	15	25	50	5	10	25	75	125
*Arctii lappae folium*	H_2_O	19.14 ± 1.29	30.00 ± 1.81	63.68 ± 4.12	90.15 ± 1.91	91.43 ± 0.86	4.62 ± 2.04	10.98 ± 1.44	25.49 ± 3.00	53.6 ± 3.54	96.13 ± 2.31 ^##^	27.81 ± 2.02	47.42 ± 3.40	64.67 ± 5.46	77.55 ± 5.46	83.24 ± 3.57
		[SC_50_ = 32.99 ± 2.44]					[SC_50_ = 17.92 ± 1.84]					[SC_50_ = 26.52 ± 5.22]				
	EtOH	15.36 ± 0.64	29.06 ± 1.71	55.22 ± 2.51	91.69 ± 0.76	92.21 ± 0.72	6.70 ± 2.76	13.75 ± 2.65	30.05 ± 4.38	94.72 ± 3.19 ^##^	98.90 ± 1.03 ^##^	22.58 ± 1.54	38.93 ± 2.91	61.51 ± 3.98	75.79 ± 4.46	76.38 ± 5.48
		[SC_50_ = 36.17 ± 1.62]					[SC_50_ = 12.28 ± 1.01]					[SC_50_ = 35.19 ± 6.30]				
*Arctii lappae radix*	H_2_O	5.68 ± 0.75	22.11 ± 5.26	22.40 ± 5.35	29.55 ± 4.02	43.81 ± 3.71	5.29 ± 1.26	6.26 ± 0.60	8.22 ± 2.94	10.92 ± 2.07	19.68 ± 2.77	15.83 ± 1.39	19.40 ± 1.59	29.79 ± 2.68	38.07 ± 3.09	52.64 ± 4.73
		[SC_50_ = NA]					[SC_50_ = NA]					[SC_50_ = NA]				
	EtOH	3.60 ± 1.15	6.63 ± 1.37	16.92 ± 2.53	34.75 ± 2.02	50.33 ± 3.23	5.27 ± 3.40	9.22 ± 3.49	11.93 ± 3.85	11.14 ± 2.69	13.33 ± 3.14	12.60 ± 1.15	14.30 ± 0.74	18.87 ± 1.47	35.32 ± 2.76	44.36 ± 2.90
		[SC_50_ = NA]					[SC_50_ = NA]					[SC_50_ = NA]				
*Arctii mini folium*	H_2_O	6.61 ± 2.06	23.09 ± 2.20	37.37 ± 7.94	40.94 ± 5.66	40.73 ± 3.21	2.21 ± 0.86	3.55 ± 1.50	2.72 ± 1.59	5.30 ± 1.88	9.71 ± 2.30	12.96 ± 1.62	18.37 ± 1.60	40.74 ± 3.32	54.39 ± 0.85	62.71 ± 2.10
		[SC_50_ = NA]					[SC_50_ = NA]					[SC_50_ = 90.22 ± 9.76]				
	EtOH	8.51 ± 1.63	11.60 ± 1.33	21.60 ± 1.95	43.60 ± 1.56	69.21 ± 2.38	NA	3.38 ± 1.49	4.22 ± 0.08	9.56 ± 0.70	18.71 ± 4.78	19.44 ± 0.93	24.80 ± 1.29	30.52 ± 2.06	43.99 ± 2.44	59.23 ± 3.41
		[SC_50_ = 119.11 ± 11.94]					[SC_50_ = NA]					[SC_50_ = 140.54 ± 32.80]				
*Arctii mini radix*	H_2_O	15.55 ± 1.82	16.82 ± 0.99	25.63 ± 1.24	60.70 ± 3.02	73.85 ± 5.16	7.05 ± 1.67	9.97 ± 1.82	15.52 ± 1.41	19.35 ± 2.44	52.79 ± 6.57	36.09 ± 0.65	43.69 ± 1.00 ^#^	57.90 ± 2.68	63.93 ± 2.78	75.87 ± 6.50
		[SC_50_ = 79.23 ± 11.74]					[SC_50_ = 116.35 ± 56.73]					[SC_50_ = 30.14 ± 4.77]				
	EtOH	8.56 ± 1.22	18.42 ± 1.14	38.44 ± 2.27	67.66 ± 3.08	84.58 ± 0.70	3.67 ± 1.31	6.94 ± 2.50	11.44 ± 1.51	22.87 ± 2.69	42.76 ± 4.51	44.85 ± 2.81	51.96 ± 4.52 ^#^	61.08 ± 3.56	62.08 ± 3.14	71.98 ± 2.18
		[SC_50_ = 58.43 ± 3.79]					[SC_50_ = NA]					[SC_50_ = 16.99 ± 7.31]				
*Calendulae flos*	H_2_O	8.34 ± 0.76	20.51 ± 3.04	15.74 ± 1.40	44.09 ± 2.79	55.12 ± 1.94	7.00 ± 1.11	12.82 ± 1.3	15.10 ± 3.59	20.37 ± 1.96	25.71 ± 3.90	8.89 ± 0.59	28.80 ± 3.70	24.85 ± 2.62	35.80 ± 3.18	53.23 ± 4.17
		[SC_50_ = NA]					[SC_50_ = NA]					[SC_50_ = NA]				
	EtOH	7.85 ± 0.43	7.53 ± 0.97	16.71 ± 1.83	33.32 ± 1.52	49.51 ± 2.98	7.70 ± 4.09	8.82 ± 1.79	10.25 ± 2.97	10.70 ± 2.78	39.75 ± 2.26	4.82 ± 0.54	7.13 ± 0.52	7.79 ± 0.18	14.01 ± 0.25	29.24 ± 2.00
		[SC_50_ = NA]					[SC_50_ = NA]					[SC_50_ = NA]				
*Centaureae flos*	H_2_O	9.90 ± 1.25	13.84 ± 0.37	33.79 ± 3.99	66.83 ± 2.70	73.41 ± 4.88	3.23 ± 0.09	4.56 ± 2.35	7.64 ± 3.84	11.25 ± 1.70	22.23 ± 4.81	16.35 ± 0.70	23.39 ± 2.21	32.47 ± 2.45	54.77 ± 5.32	67.64 ± 6.4
		[SC_50_ = 71.76 ± 10.23]					[SC_50_ = NA]					[SC_50_ = 86.31 ± 24.02]				
	EtOH	6.56 ± 1.02	12.03 ± 0.62	26.68 ± 2.12	54.26 ± 3.09	77.69 ± 5.25	7.07 ± 2.47	4.83 ± 2.37	8.41 ± 3.36	12.88 ± 2.73	24.54 ± 3.67	22.38 ± 1.6	25.00 ± 1.35	26..39 ± 1.36	38.18 ± 1.70	53.07 ± 3.57
		[SC_50_ = 83.66 ± 11.56]					[SC_50_ = NA]					[SC_50_ = NA]				
*Galii aparinae herba*	H_2_O	10.93 ± 0.75	15.97 ± 1.00	35.1 ± 2.14	78.6 ± 4.74	80.47 ± 1.34	6.78 ± 2.60	6.5 ± 1.33	15.64 ± 3.78	20.79 ± 1.94	34.20 ± 4.83	23.51 ± 1.46	34.7 ± 2.68	40.24 ± 4.55	64.67 ± 5.64	75.09 ± 3.02
		[SC_50_ = 56.93 ± 6.94]					[SC_50_ = NA]					[SC_50_ = 29.80 ± 6.83]				
	EtOH	11.40 ± 0.65	14.56 ± 1.88	32.07 ± 1.88	77.44 ± 3.40	85.54 ± 2.24	4.75 ± 2.20	9.06 ± 2.94	16.44 ± 3.87	18.12 ± 5.79	41.48 ± 4.38	30.11 ± 3.40	39.03 ± 3.98	46.98 ± 4.65	63.72 ± 5.23	72.81 ± 2.89
		[SC_50_ = 56.40 ± 4.40]					[SC_50_ = NA]					[SC_50_ = 24.92 ± 7.89]				
*Millefolii herba*	H_2_O	18.66 ± 4.55	22.8 ± 9.93	44.63 ± 2.48	80.48 ± 3.07	87.36 ± 2.46	8.58 ± 3.02	9.88 ± 3.81	22.94 ± 3.70	35.15 ± 1.76	35.76 ± 5.43	18.97 ± 1.12	24.04 ± 2.84	51.41 ± 4.94	82.44 ± 2.94	85.71 ± 1.58
		[SC_50_ = 44.39 ± 7.85]					[SC_50_ = NA]					[SC_50_ = 41.41 ± 4.35]				
	EtOH	21.42 ± 3.60	30.94 ± 1.87	55.17 ± 1.00	90.53 ± 1.26	92.1 ± 1.36	10.26 ± 2.53	12.76 ± 3.23	17.05 ± 2.87	26.04 ± 5.07	69.09 ± 4.30 ^#^	27.37 ± 3.43	45.66 ± 4.62	64.29 ± 4.49	75.41 ± 5.14	77.80 ± 6.11
		[SC_50_ = 33.76 ± 2.58]					[SC_50_ = 48.15 ± 13.67]					[SC_50_ = 28.63 ± 7.48]				
*Sambuci nigrae folium*	H_2_O	15.40 ± 2.02	15.45 ± 2.19	47.94 ± 3.92	83.41 ± 4.40	86.73 ± 3.32	7.85 ± 5.63	11.13 ± 1.65	16.40 ± 3.16	21.37 ± 4.31	40.58 ± 5.6	23.23 ± 2.49	41.21 ± 3.22	51.93 ± 3.5	71.00 ± 3.94	79.67 ± 2.36
		[SC_50_ = 46.09 ± 5.30]					[SC_50_ = NA]					[SC_50_ = 38.14 ± 6.30]				
	EtOH	14.80 ± 0.87	32.25 ± 3.00	53.83 ± 2.46	89.92 ± 1.62	90.29 ± 1.15	4.68 ± 1.91	4.00 ± 2.66	18.13 ± 2.63	37.46 ± 5.71	94.81 ± 2.29 ^##^	31.32 ± 3.10	34.41 ± 2.84	43.07 ± 3.05	67.35 ± 2.01	75.83 ± 1.79
		[SC_50_ = 36.49 ± 2.44]					[SC_50_ = 23.26 ± 2.92]					[SC_50_ = 44.64 ± 7.22]				
*Serpylli herba*	H_2_O	22.76 ± 4.11	47.97 ± 3.35 ^##^	84.89 ± 3.49 ^##^	88.82 ± 1.81	86.33 ± 2.88	13.78 ± 2.12	23.05 ± 3.42 ^##^	47.09 ± 3.07 ^##^	82.22 ± 2.66 ^#^	96.46 ± 2.70 ^##^	40.62 ± 3.92 ^#^	46.39 ± 4.02	64.94 ± 4.52	76.09 ± 3.07	76.36 ± 2.52
		[SC_50_ = 22.31 ± 3.48]					[SC_50_ = 10.87 ± 1.04]					[SC_50_ = 20.39 ± 6.10]				
	EtOH	39.12 ± 2.79 ^##^	49.54 ± 0.88 ^##^	88.11 ± 6.06 ^##^	91.93 ± 3.46	92.13 ± 2.55	14.76 ± 2.34	23.48 ± 2.82 ^##^	23.48 ± 2.82	85.10 ± 3.31 ^#^	99.17 ± 1.11 ^##^	39.49 ± 3.34 ^#^	59.53 ± 2.32 ^##^	70.48 ± 2.70	74.19 ± 5.64	74.60 ± 4.94
		[SC_50_ = 15.02 ± 2.09]					[SC_50_ = 9.41 ± 0.71]					[SC_50_ = 13.60 ± 3.45]				
*Taraxaci herba*	H_2_O	9.91 ± 1.12	21.47 ± 1.35	45.61 ± 2.14	79.21 ± 4.77	84.62 ± 3.48	5.34 ± 2.32	8.25 ± 2.18	31.65 ± 3.49	72.88 ± 3.29	97.64 ± 2.17 ^##^	22.23 ± 1.68	24.63 ± 2.55	48.52 ± 2.64	71.49 ± 3.06	76.12 ± 3.19
		[SC_50_ = 48.91 ± 4.78]					[SC_50_ = 14.69 ±1.25]					[SC_50_ = 49.00 ± 6.37]				
	EtOH	22.08 ± 1.93	23.97 ± 1.49	49.35 ± 3.14	87.62 ± 3.17	88.88 ± 2.63	2.81 ± 1.27	14.81 ± 2.73	47.44 ± 6.68 ^##^	73.16 ± 4.74	95.83 ± 3.29 ^##^	19.62 ± 2.35	25.87 ± 2.47	64.60 ± 3.18	64.60 ± 3.18	69.74 ± 3.29
		[SC_50_ = 38.63 ± 3.57]					[SC_50_ = 13,10 ± 1,59]					[SC_50_ = 60.59 ± 10.09]				
*Urticae herba*	H_2_O	19.49 ± 2.38	23.00 ± 1.35	50.29 ± 3.45	82.57 ± 1.51	78.85 ± 3.67	7.61 ± 1.40	11.55 ± 4.01	15.35 ± 2.20	23.82 ± 3.39	34.54 ± 2.65	21.75 ± 0.95	24.95 ± 1.25	35.42 ± 2.15	60.50 ± 4.45	76.15 ± 4.29
		[SC_50_ = 44.15 ± 4.61]					[SC_50_ = NA]					[SC_50_ = 63.75 ± 10.52]				
	EtOH	15.78 ± 2.52	17.97 ± 0.56	37.98 ± 1.62	83.14 ± 5.46	89.81 ± 0.91	7.35 ± 3.16	10.56 ± 4.12	21.59 ± 3.97	34.13 ± 4.67	61.82 ± 2.64 ^#^	10.23 ± 0.70	18.94 ± 1.48	22.43 ± 2.18	47.51 ± 2.14	67.28 ± 3.73
		[SC_50_ = 47.54 ± 3.79]					[SC_50_ = 45.97 ± 9.99]					[SC_50_ = 112.91 ± 17.59]				
*Violae herba*	H_2_O	10.32 ± 1.25	14.89 ± 2.44	15.85 ± 2.22	32.51 ± 8.35	55.55 ± 3.38	11.33 ± 2.05	13.35 ± 2.09	15.37 ± 1.78	23.60 ± 2.64	34.42 ± 2.20	13.18 ± 0.82	22.39 ± 2.25	33.53 ± 3.00	41.86 ± 3.37	58.41 ± 3.80
		[SC_50_ = NA]					[SC_50_ = NA]					[SC_50_ = 140.39 ± 37.08]				
	EtOH	10.57 ± 1.30	16.61 ± 0.82	23.40 ± 1.80	47.54 ± 0.63	66.06 ± 0.69	7.29 ± 0.86	5.40 ± 0.84	10.44 ± 1.32	10.44 ± 1.32	22.17 ± 1.64	15.22 ± 0.92	24.96 ± 2.32	24.85 ± 1.16	28.94 ± 1.98	50.71 ± 3.81
		[SC_50_ = 115.99 ± 5.57]					[SC_50_ = NA]					[SC_50_ = NA]				

**Table 3:** Sum of polyphenols determined by the Folin–Ciocalteu method. ^##^—extracts with the highest content of polyphenols (*p* < 0.001).

Extract	Aqueous (% ± SD)	70% Ethanolic (% ± SD)
*Arctii lappae folium*	8.34 ± 0.60	11.74 ± 1.14
*Arctii lappae radix*	2.60 ± 0.15	2.57 ± 0.24
*Arctii mini folium*	3.11 ± 0.77	4.67 ± 0.72
*Arctii mini radix*	9.25 ± 1.54	8.20 ± 0.89
*Calendulae flos*	4.95 ± 0.51	5.15 ± 0.60
*Centaureae flos*	6.07 ± 0.85	6.82 ± 0.87
*Galii aparinae herba*	8.01 ± 1.14	6.78± 1.33
*Millefolii herba*	9.90 ± 0.82	13.61 ± 1.87
*Sambuci nigrae folium*	10.55 ± 0.77	9.78 ± 0.66
*Serpylli herba*	20.61 ± 1.13 ^##^	21.25 ± 2.90 ^##^
*Taraxaci herba*	10.05 ± 0.93	12.46 ± 1.77
*Urticae herba*	8.64 ± 1.03	8.36 ± 1.08
*Violae herba*	6.02 ± 1.23	6.68 ± 1.44

**Table 4:** The MS2 data of compounds detected in *Millefolii herba* ethanolic extract (K EtOH), *Serpylli herba* ethanolic extract (Th EtOH), and *Arctii lappae folium* ethanolic extract (All EtOH). NL—neutral loss detected corresponding to the cleavage of sugar or phenolic acid.

No.		Rt (min)	Compound	Max. UV (nm)	(M − H)^−^ *m*/*z*	MS^2^ ions *m*/*z*	NL Detected Amu
K EtOH	1	8.1	Caffeoylquinic acid	216	353	191	-
	2	15.8	Quercetin *O*-hexoside	202, 332	463	301	162
	3	18.8	Luteolin *O*-hexoside Luteolin *O*-glucuronide	204, 343	447 461	285 285	162 176
	4	21.4	Dicaffeoylquinic acid	213, 329	515	353	162
	5	21.8	Dicaffeoylquinic acid Apigenin *O*-hexoside	204, 329	515 431	353 269	162 162
	6	22.9	Dicaffeoylquinic acid	213, 328	515	353	162
Th EtOH	1	22.9	Quercetin *O*-glucuronide	212, 281, 343	477	301	176
	2	26.2	Luteolin *O*-glucuronide	271, 338	461	285	176
	3	30.2	Apigenin *O*-glucuronide	267, 334	445	269	176
	4	31.4	Rosmarinic acid	197, 328	359	197	162
	5	32.6	Salvianolic acid K	198, 323	555	493, 359, 161	-
	6	34.1	Salvianolic acid (H, I or J)	198, 325	537	493, 359, 161-	-
All EtOH	1	8.0	Caffeoylquinic acid	216,326	353	191	-
	2	21.1	Dicaffeoylmaloylquinic acid	216, 328	631	515, 469, 353	162
	3	23.0	Dicaffeoylsuccinoylquinic acid	216, 329	615	515. 453, 353	162
	4	25.0	Dicaffeoylsuccinoylquinic acid	217, 328	615	515, 453, 353, 191	162
	5	26.7	Dicaffeoyldisuccinoylquinic acid	218, 329	715	553	162
	6	29.4	Tricaffeoylsuccinoylquinic acid	218, 327	777	615	162

## Data Availability

The data presented in this study are available on request from the corresponding author.

## Supplementary Materials

The following are available online at https://www.mdpi.com/article/10.3390/antiox10050698/s1, Table S1: Timetable for preparing the extracts and conducting the experiments, Figure S1: Concentration–activity curves of the most active extracts in the test of LOX inhibitory activity.
