# Supplementary material for: Screening of Antioxidative Properties and Inhibition of Inflammation-Linked Enzymes by Aqueous and Ethanolic Extracts of Plants Traditionally Used in Wound Healing in Poland
Source: Antioxidants (Basel). 2021 Apr 28;10(5):698. doi: 10.3390/antiox10050698 (PMC8146166; doi:10.3390/antiox10050698)
Supplement: Supplementary file 1 [file antioxidants-10-00698-s001.zip › antioxidants-1186063-supplementary.pdf]

antioxidants

MDPI

Supplementary Materials: Screening of Antioxidative Properties and Inhibition of Inflammation-Linked Enzymes by Aqueous and Ethanolic Extracts of Plants Traditionally Used in Wound Healing in Poland

Marta Mainka [1], Monika E. Czerwińska [1], Ewa Osińska [2], Maria Ziaja [3] and Agnieszka Bazylko [1,*]

[1] Department of Pharmacognosy and Molecular Basis of Phytotherapy, Warsaw Medical University, Banacha 1, 02-097 Warsaw, Poland; marta.rogowska@wum.edu.pl (M.M.); monika.czerwinska@wum.edu.pl (M.E.C.)
[2] Department of Vegetable and Medicinal Plants, Warsaw University of Life Sciences, Nowoursynowska 159, 02-776 Warsaw, Poland; ewa_osinska@sggw.pl
[3] Institute of Physical Culture Studies, Rzeszów University, Cicha 2a, 35-326 Rzeszów, Poland; mziaja@ur.edu.pl
* Correspondence: agnieszka.bazylko@wum.edu.pl

Table S1. Timetable for preparing the extracts and conducting the experiments.

Activity.	Start Date	End Date
UHPLC-DAD-MS^n Analysis	08-23-2017	09-12-2017
Preparation of extracts	10-14-2016	12-14-2016
Determination of extracts ability for the scavenging of DPPH	12-20-2016	01-26-2017
Determination of extracts ability for the scavenging of superoxide anion	01-31-2017	03-09-2017
Determination of extracts ability for the scavenging of hydrogen peroxide	09-14-2017	10-03-2017
Determination of extracts effect on the Lipoxygenase activity	04-27-2017	08-24-2017
Determination of extracts effect on the Hyaluronidase activity	10-26-2017	12-21-2017
Determination of total polyphenol content by colorimetric method with Folin'a Ciocalteu reagent	09-05-2017	09-05-2017

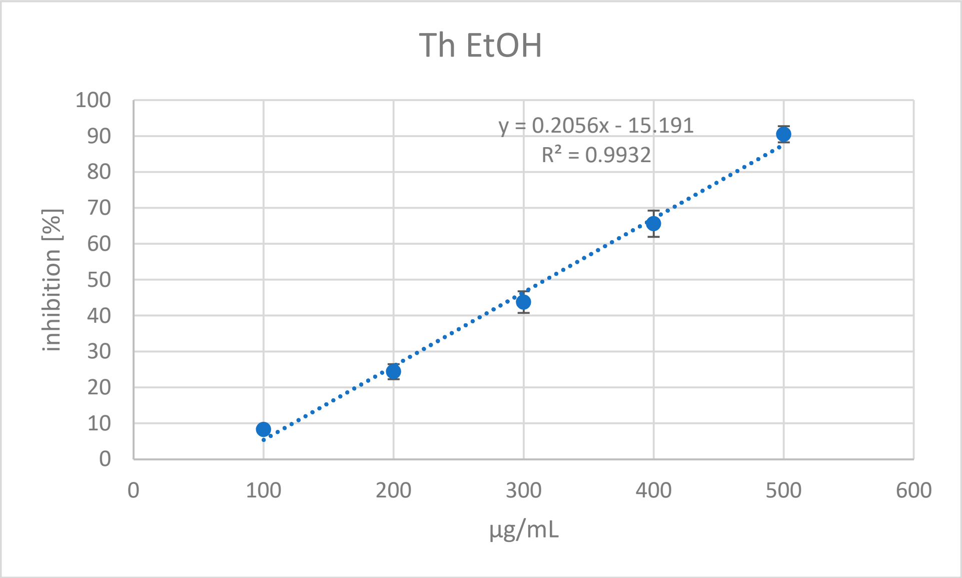

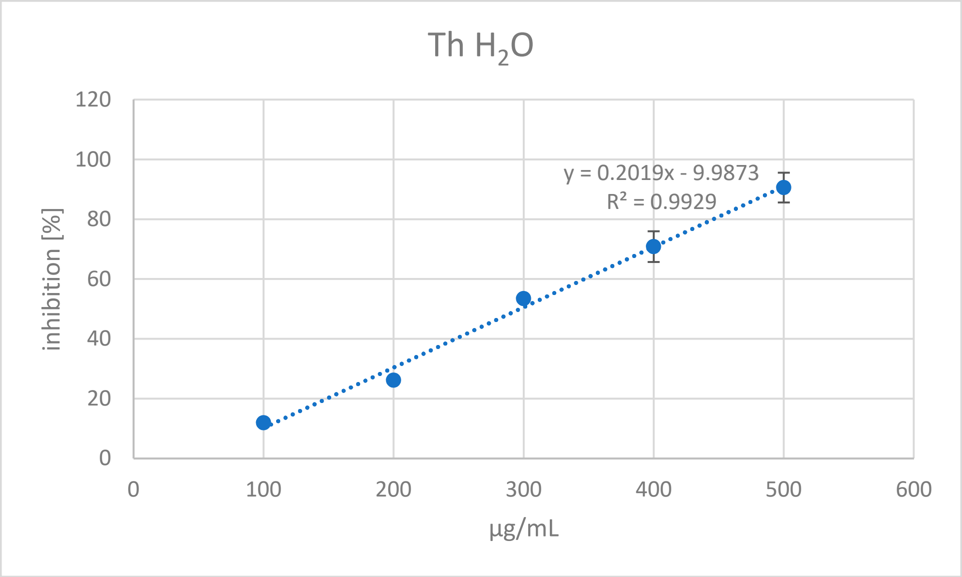
Th H2O
y = 0.2019x - 9.9873
R² = 0.9929
inhibition [%]
µg/mL
0
20
40
60
80
100
120
0
100
200
300
400
500
600

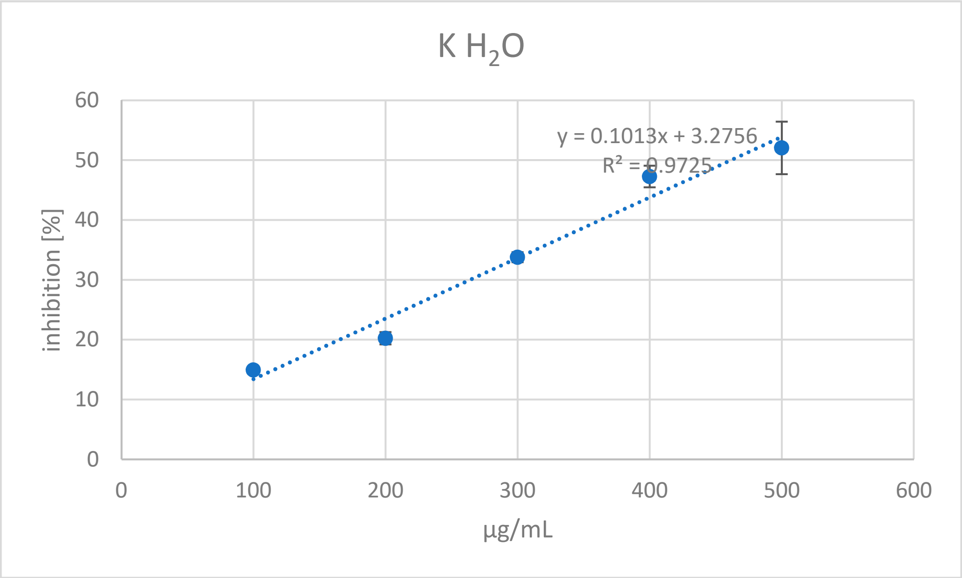
K H2O
y = 0.1013x + 3.2756
R² = 0.9725
inhibition [%]
µg/mL
0
10
20
30
40
50
60
0
100
200
300
400
500
600

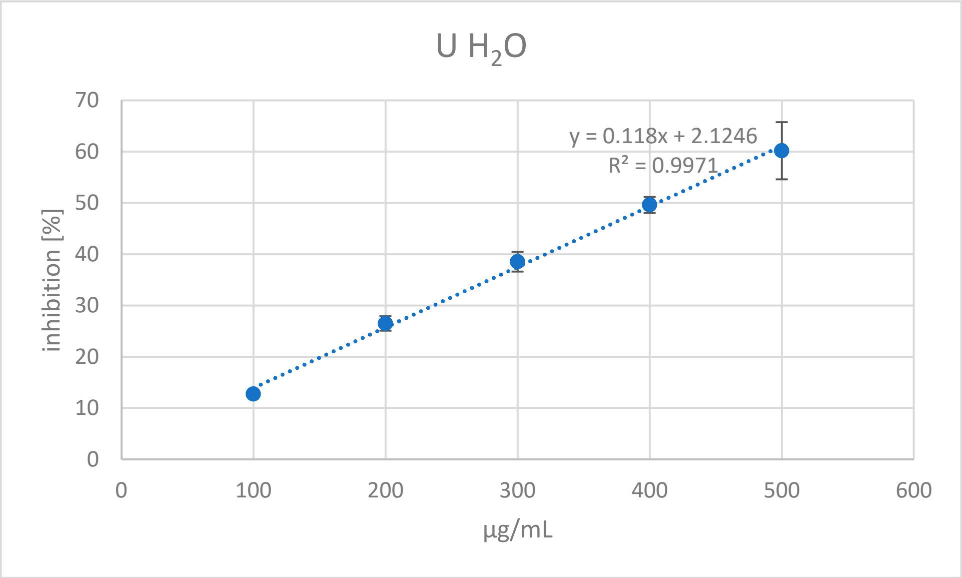
U H2O
y = 0.118x + 2.1246
R² = 0.9971
inhibition [%]
µg/mL
0
10
20
30
40
50
60
70
0
100
200
300
400
500
600

Figure S1. Concentration-activity curves of the most active extracts in the test of LOX inhibitory activity. Th EtOH – *Serpylli herba* ethanolic extract, Th H_2O – *Serpylli herba* aqueous extract, K H_2O – *Milefolli herba* aqueous extract, U H_2O – *Urticae herba* aqueous extract.
